# Evaluation of Dental Anxiety, Demographic Characteristics, and Oral Health Profiles Among Non-Physician Healthcare Undergraduate Students

**DOI:** 10.3290/j.ohpd.c_2482

**Published:** 2026-03-04

**Authors:** Gülçin Bulut, Pınar Güvenç, Hülya Erten, Bilge Cansu Uzun

**Affiliations:** a Gülçin Bulut Associate Professor, Dokuz Eylul University, Faculty of Dentistry, Department of Pediatric Dentistry, Izmir, Turkey. Study concept and design, performed the examinations, wrote the manuscript, read and approved the final manuscript.; b Pınar Güvenç Assistant Professor, Dokuz Eylul University, Faculty of Dentistry, Department of Restorative Dentistry, Izmir, Turkey. Study concept and design, performed the examinations, performed analyses, and read and approved the final manuscript.; c Hülya Erten Professor, Dokuz Eylul University, Faculty of Dentistry, Department of Restorative Dentistry, Izmir, Turkey. Study concept and design, administered surveys to the study population, proofread the manuscript, and read and approved the final manuscript.; d Bilge Cansu Uzun Assistant Professor, Dokuz Eylul University, Faculty of Dentistry, Department of Periodontology, Izmir, Turkey. Study concept and design, administered surveys to the study population, and read and approved the final manuscript.

**Keywords:** dental anxiety, healthcare students, MDAS, oral-dental health, STAI.

## Abstract

**Purpose:**

To assess the relationship between dental anxiety and sociodemographic factors, oral health status, and oral health behaviors among non-physician healthcare (NPH) undergraduate students.

**Materials and Methods:**

This study included students from the Faculty of Physical Therapy and Rehabilitation (PTR), the Faculty of Nursing, and the Vocational School of Healthcare (VSH). Dental anxiety levels were measured using the Modified Dental Anxiety Scale (MDAS) and Spielberg’s State-Trait Anxiety Inventory (STAI). The students completed a questionnaire regarding demographic characteristics, living, and oral hygiene habits. Oral health status was evaluated through clinical examination using the DMFT Index and the Oral Hygiene Index (OHI). Statistical analyses were performed using χ^2^, Kruskal-Wallis, and Mann-Whitney tests (p < 0.05).

**Results:**

A total of 844 students were included in the study. The average MDAS, STAI-1 and STAI-2 scores were 10.80±4.35, 41.04±6.42, and 47.06±6.75, respectively. Seven percent of the students experienced high anxiety. Females had higher MDAS and STAI-2 scores (p<0.01; p<0.01, respectively). No statistically significant differences were found in MDAS levels based on DMFT and OHI scores. Non-smokers have higher MDAS anxiety levels (p=0.002), however, lower STAI-1 scores (p<.001). MDAS and STAI-1 scores regarding family income exhibited statistically significant differences (p<0.005; p<0.005, respectively). No statistically significant difference was found between first- and final-year students regarding anxiety levels (p=0.324). Students who had regular dental visits had lower MDAS scores (p<0.001).

**Conclusion:**

Female students and those from low-income families tend to have higher dental anxiety. Regular dental visits help reduce anxiety among NPH students. Smoking also contributes to dental anxiety.

Dental anxiety is an excessive psychological stress response triggered by the thought of visiting the dentist for preventive care or dental procedures. It is a global issue, with prevalence rates ranging from 6.3% to 41.7% worldwide. According to previous studies, it affects individuals of all genders, ages, educational backgrounds, and social status.^4^

Dental anxiety is a multifactorial condition that may limit or entirely prevent individuals from completing dental treatment, potentially worsening existing oral health.^28^ Initially manageable dental issues can progress into more severe conditions due to delayed or missed appointments, requiring more complex interventions. High levels of dental anxiety are associated with poor oral hygiene, which accelerates the progression of dental issues and often requires extensive treatments.^4^ When these situations become more advanced, individuals frequently experience widespread periodontal diseases, higher numbers of caries, fewer restored teeth, and overall poor oral health.^6,33^

Several tools have been developed to assess dental anxiety. Among them, the Modified Dental Anxiety Scale (MDAS), introduced by Humphris et al in 1995,^16^ is widely used for its simplicity, consistent scoring, and ease of administration without increasing patient anxiety. Another common instrument is the State-Trait Anxiety Inventory (STAI), created by Spielberger et al^5^ in 1970, which measures both transient feelings of situation-specific anxiety (state) and a general tendency toward anxiety (trait). Its use in dental practice supports effective treatment planning, anxiety management, and improved patient satisfaction.^26^

Numerous research on dental anxiety performed among undergraduates have predominantly focused on dental and medical students.^3,10,25,31^ However, undergraduate healthcare student populations, in fields such as nursing, physiotherapy, and other healthcare disciplines, have been less represented in these studies. Additionally, while the oral health status of general undergraduate students has been the object of investigation in various universities worldwide,^8,14,40^ reports regarding the DMFT score and periodontal health among non-dental students are very limited.^15,29^ Understanding the oral health status of non-physician healthcare students and its association with dental anxiety is essential, particularly in examining how demographic factors, living conditions, and oral hygiene habits influence this anxiety. Since these students interact directly with patients, their own attitudes toward oral health can shape future practices and play a key role in motivating patients to seek dental care.

The aim of this study was to investigate the association between dental anxiety and sociodemographic characteristics, as well as oral health parameters, specifically dental and periodontal status, among non-dental and non-medical healthcare students at Dokuz Eylul University in Izmir, Turkey.

## MATERIALS AND METHODS

### Population and Study Design 

This cross-sectional study was conducted on healthcare students at Dokuz Eylul University during the 2021-2022 academic year, excluding those in medical and dental faculties. The study included students from the Faculty of Physical Therapy and Rehabilitation (PTR), the Faculty of Nursing, and the Vocational School of Healthcare (VSH). A sample size of 402 was determined, based on a two-sided 95% confidence interval with an impact width of d=0.100 when the sample proportion is 0.500.

The study was designed to perform a dental examination, including the assessment of dental and periodontal conditions, after participants completed a questionnaire in a face-to-face setting in the classroom. The questionnaires were collected immediately after they were filled out. Students who were absent on the day of the procedure, those who did not participate in at least one of these activities, and those who completed the survey partially were excluded from the study.

Dokuz Eylul University Faculty of Medicine approved the study protocol by the Non-Interventional Research Ethics Committee (2022/05-06). Students were informed about the research topic, and each volunteer student who consented to participate signed a written informed consent form.

Out of 1610 undergraduate students attending these faculties or schools, 844 students who met the criteria enrolled in the study voluntarily.

### Questionnaires

Students completed paper-based surveys during class hours with permission from the lecturers under the supervision of two dentists in their classrooms (HE and CU). The survey form consists of two parts. In the first part, the questionnaire includes 10 questions covering demographic features, including age, gender, grade level, parental education, and family income, as well as oral hygiene and living habits, including smoking, dental visit frequency, previous dental treatments, oral hygiene routines, and self-reported oral health status. The questionnaire used closed-ended questions with multiple-choice answers. Family income was calculated based on the income distribution table published by the Turkish Statistical Institute, using current data at the time the study was conducted.^36^ Regular dental check-ups were defined as having regular visits to the dentist for approximately one year (±1 month), excluding complaints and ongoing treatment.^24^

In the second part, the Modified Dental Anxiety Scale (MDAS) and the State-Trait Anxiety Inventory (STAI I-II) were used to evaluate participants’ dental anxiety levels.

### Dental Anxiety Score

The MDAS consists of five questions, each rated on a 5-point Likert scale. The response options range from (1) “not anxious” to (5) “extremely anxious”, with the intermediate options being (2) “slightly anxious”, (3) “fairly anxious”, and (4) “very anxious”. Therefore, each question is scored between 1 and 5; the maximum score on the entire scale is 25, and the minimum score is 5. MDAS scores were classified as 5-9, 10-18, and ≥ 19, with the definitions being less anxiety, moderate anxiety, and high dental anxiety, respectively. The questions are as follows: 1. If you had to visit the dentist tomorrow, how would you feel about it? 2. While waiting for your turn in the dental office, how would you feel? 3. How would you feel if you knew your teeth were going to be drilled? 4. How would you feel if you knew that your teeth would be scaled and polished? 5. How would you feel if you knew that a local anesthetic injection would be administered over an upper back tooth? MDAS has also been tested and validated for use in Turkish patients,^24^ thus ensuring its reliability in measuring dental anxiety.

The STAI has 40 items divided equally between the S-Anxiety (State Anxiety-STAI-1) and T-Anxiety (Trait Anxiety-STAI-2) subscales, with 20 items each. Responses for the STAI-1 scale measure the current intensity of feelings “at this moment” using these options: 1) not at all, 2) somewhat, 3) moderately so, and 4) very much so. Responses for the STAI-2 scale assess the frequency of feelings “in general,” with options: 1) almost never, 2) sometimes, 3) often, and 4) almost always. The total score ranges from 20 to 80, with 80 being the highest and 20 the lowest. A higher total score indicates higher anxiety levels. The inventory was adapted and standardized into Turkish by Oner and Le Compte.^24^

### Oral Examination

The oral examination was conducted by two dentists (GB and PG) using a dental probe and a mirror, with the assistance of an overhead lamp in the classroom. Caries status was determined according to DMFT index criteria defined by WHO.^37^

Periodontal status was assessed using the simplified oral hygiene index (OHI-S), which was based on a combination of debris index (DI) and calculus index (CI).^12^ The examinations were carried out on the buccal and lingual surfaces of six index teeth (i.e., teeth 16, 11, 25, 36, 32, 45) by recording the scores for the amount of debris or calculus. The scores were summed and then divided by the total number of surfaces to determine the OHI-S value for each individual.

Twenty patients from the same age group who visited the clinic 14 days prior to the study were randomly selected and evaluated by two researchers at two different clinics. Seven days after the initial evaluation, the same patients were contacted and re-evaluated by the same researchers. The Cohen Kappa value was 0.88 for intra-observer agreement and 0.80 for inter-observer agreement.

### Statistical Analysis

Statistical analyses were conducted using IBM SPSS Statistics for Windows, Version 25.0 (IBM; Armonk, NY, USA). The normality of numerical variables was evaluated with the Shapiro-Wilk test. Association analysis was performed using non-parametric methods, as the data did not follow a normal distribution. Group comparisons included the Mann-Whitney U-test, χ^2^ test, and Kruskal-Wallis test. Post-hoc pairwise comparisons were made using Dunn’s test with Bonferroni correction after the Kruskal-Wallis test. A p-value <0.05 was considered statistically significant.

## RESULTS

Out of 844 enrolled undergraduate students, all completed the study, resulting in a response rate of 100%. The study population included students from three faculties or schools: PTR (n=236; 27.96%), Nursing (n=319; 37.79%), and VSH (n=289; 34.24%). Demographic features as well as daily living and oral hygiene habits of the study population are presented in Table 1.

**Table 1:** Demographic features, living, and oral hygiene habits of the study population

		n (%)
Age		21.68 ± 1.77*
Gender	Female	601 (71.2)
	Male	243 (28.8)
Family income	Low	420 (49.8)
	Medium	300 (35.5)
	High	124(14.7)
Parental education	Illiterate	31 (3.7)
	Literate	158 (18.8)
	8-year education	237(28.1)
	Highschool	266 (31.5)
	University	152 (18)
Smoking habit	None	573 (68)
	<1 pack a day	132 (15.6)
	>1 pack a day	59 (7)
	Smoked and quit	80 (9.4)
Frequency of toothbrushing	Once a day	257(30.5)
	Twice a day	551(65.3)
	1-2 times a week	27 (3.2)
	Do not brush	60 (1.1)
Self-reported oral hygiene	Very good (entirely healthy)	52(6.2)
	Good (healthy)	287(34)
	Moderate	418(49.6)
	Bad (poor)	75(8.8)
	Very bad (extremely poor)	12(1.4)
Self-reported oral health	Very good (entirely healthy)	45(5.2)
	Good (healthy)	243(28.3)
	Moderate	418(49.5)
	Bad (poor)	116(13.7)
	Very bad (extremely poor)	24 (2.8)
Frequency of dental visits	Never	55 (6.5)
	When a problem occurs	672 (79.6)
	Regularly	117 (13.9)
Treatments performed in a dental clinic		
Filling	None	377 (44.7)
	At least one	467 (55.3)
Extraction	None	581 (68.8)
	At least one	263 (31.2)
Other	None	536 (63.50)
	At least one	308(36.50)
*Mean ± standard deviation.		

**Fig 1:**
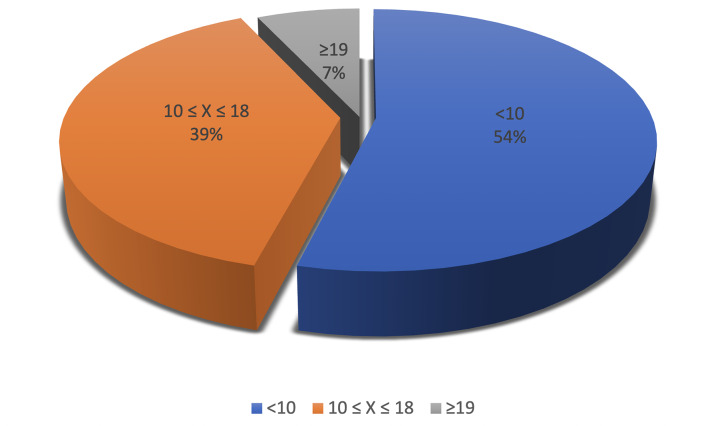
Distribution of students by MDAS levels.

The distribution of students by MDAS levels is shown in Fig 1. The average MDAS score for all students was 10.80±4.35 with a median of 10.0 (range 5-25). The highest percentage of ‘extremely anxious’ responses occurred at MDAS 3 with 6.8% and at MDAS 5 with 6.2%. Student MDAS scores ranged from 5 to 25, with only 0.9% (n=8) of students reaching the maximum score of 25. The mean ± SD (median; min-max) STAI-1 and STAI-2 scores for the study population were 41.04±6.42 (40; 23-80) and 47.06±6.75 (46; 28-80), respectively.

Regarding gender, female students had higher MDAS and STAI-2 scores than male students (p<0.01; p<0.01, respectively); however, males scored higher on the STAI-1 index (p<0.01, Mann-Whitney U-Test) (Table 2).

**Table 2:** Comparison of anxiety levels, DMFT, and OHI scores according to gender

	Female	Male	p
MDAS Mean ± SD (median; min-max)	11.45±4.36 (11; 5–25)	9.35±4.10 (8; 5–25)	<0.001*
STAI-1 Mean ± SD (median; min-max)	40.29±6.33 (40; 23–80)	43.04±6.39 (43; 28–64)	<0.001*
STAI-2 Mean ± SD (median; min-max)	47.76±6.837 (47; 32–80)	45.40±6.511 (45; 28–80)	<0.001*
DMFT	3.23±2.499 (3;0–13)	3.09±2.559 (3;0–13)	0.393
OHİ	1.55±1.63 (0.99;0–6.16)	1.79±1.79 (1;0–6.16)	0.112
* p<0.05; statistically significant, Mann-Whitney U-test.			

The students’ average DMFT and OHI scores were 3.23±2.50 (2; 0-13), and 1.60±1.63 (1; 0-6.16), respectively. No statistically significant differences were observed in DMFT and OHI scores based on gender (p=0.393; p=0.112, respectively, Mann-Whitney U-test) (Table 2). As shown in Table 3, no statistically significant differences were found in MDAS levels according to DMFT and OHI scores.

**Table 3:** Comparison of DMFT and OHI scores according to the MDAS levels

	MDAS <10	MDAS 10-18	MDAS ≥19	p
DMFT	3.27±2.63 (3.0; 0–13)	3.10±2.44 (3.0; 0–13)	3.32±2.27 (3.0; 0–9)	0.679
OHI	1.70±1.69 (1.0; 0–6.16)	1.55±1.68 (0.83; 0–6.16)	1.60±1.68 (0.99; 0–5.66)	0.262
*p<0.05; statistically significant, Kruskal-Wallis test.				

The differences in anxiety levels based on family income and parental education levels are presented in Table 4. MDAS and STAI-1 scores of the students regarding family income exhibited statistically significant differences (p<0.005; p<0.005, respectively, Kruskal-Wallis). While students from low-income families had higher anxiety levels than those from high-income families in MDAS, higher anxiety levels were observed in students from high-income families compared to low-income families in STAI-1 (p=0.004; p=0.003, respectively, Dunn’s test with Bonferroni corrections). No statistically significant differences were observed in the relationships between MDAS, STAI-1, and STAI-2 scores and educational level (p=0.105; p=0.923; p=0.620, respectively; Kruskal-Wallis test).

**Table 4:** Comparison of anxiety levels among the family income and parental education levels

		MDAS Mean ± SD (median; min-max)	p	STAI-1 Mean ± SD (median; min-max)	p	STAI-2 Mean ± SD (median; min-max)	p
**Parents**							
Family income	Low	11.2±4.449 (10; 5–25)^a^	0.005*	40.59±6.550 (40; 23–80)^b^	0.005*	47.18±6.868 (46; 32–80)	0.504
	Medium	10.85±4.559 (10; 5–25)		40.99±5.957 (40; 28–64)		46.66±6.316 (46; 28–68)	
	High	9.62±3.547 (9; 5–19)^a^		42.98±6.924 (41.50; 31–63)^b^		47.77±7.791 (47; 34–80)	
**Education**							
	Illiterate	10.23±4.137 (10; 5–20)	0.105	41.74±7.620 (40; 31–64)	0.923	46.45±6.627 (46; 38–72)	0.620
	Literate	11.49±4.328 (11; 5–24)		41.55±7.060 (41; 28–80)		47.49±7.138 (32–80)	
	8–year education	10.62±4.197 (10; 5–25)		40.98±6.158 (41; 25–62)		46.81±6.369 (46; 32–78)	
	Highschool	10.95±4.541 (10; 5–25)		41.13±6.656 (40; 23–63)		46.94±7.057 (46; 32–80)	
	University	10.45±4.529 (9.50; 5–25)		40.53±5.569 (41; 28–54)		47.47±6.858 (47; 28–67)	
*Statistically significant at p<0.05. A statistically significant difference was observed between groups with the same lowercase superscript letters (Dunn’s Test with Bonferroni correction); a: low vs high, p=0.004; b: low vs high, p=0.003.							

The students’ living and oral hygiene habits, as well as their correlation with MDAS, STAI-1, and STAI-2, are presented in Table 5. In this study, 67.8% of the students reported not smoking. There were statistically significant differences in MDAS and STAI-1 scores regarding smoking (p=0.002; p<.001, respectively, Kruskal-Wallis). MDAS anxiety levels were higher in students who did not smoke compared to those who smoked less than one pack a day (p=0.002, Dunn’s test with Bonferroni corrections). STAI-1 scores were higher in students who smoked more than one pack a day than in those who did not smoke (p<.001, Dunn’s test with Bonferroni corrections). The majority of the students (65.3%) reported brushing their teeth twice a day. Regarding the frequency of toothbrushing, a statistically significant difference was detected in STAI-1 scores, while no correlations were found with MDAS and STAI-2 scores (p=0.003; p=0.290; p=0.275, respectively, Kruskal-Wallis test). STAI-1 anxiety scores were higher in students who did not brush their teeth compared to those who brushed once a day, twice a day, and 1-2 times a day (p=0.002; p=0.002; p=0.028, respectively, Dunn’s test with Bonferroni corrections). MDAS and STAI-1 scores of self-reported oral hygiene showed a significant difference (p=0.022; p<.001, respectively, Kruskal-Wallis); however, in pairwise comparisons with Dunn’s test, MDAS scores showed no statistically significant differences among the five subgroups. Similarly, the relationships between MDAS and STAI-1 scores and self-reported oral health were statistically significant (p=0.005; p=0.001, respectively, Kruskal-Wallis Test). Less anxiety was found in students who reported “very good” compared to those who reported “moderate” or “very bad” oral health (p=0.036; p=0.009, respectively, Dunn’s test with Bonferroni corrections). Conversely, higher anxiety was observed in students reporting “very good” oral health compared to those reporting “moderate” or “bad” oral health (p=0.006; p=0.039, respectively, Dunn’s test with Bonferroni corrections). There were no statistically significant differences in MDAS, STAI-1, and STAI-2 scores regarding treatments performed in a dental clinic (Table 4).

**Table 5:** Evaluation of MDAS, STAI-1, and STAI-2 scores according to living and oral hygiene habits

		n/844 (%)	MDAS Mean ± SD (median; min-max)	p	STAI-1 Mean ± SD (median; min-max)	p	STAI-2 Mean ± SD (median; min-max)	p
Smoking habit	None	573 (68)	11.15±4.301 (10; 5–25)^a^	0.002*	40.60±6.530 (40; 23–80)^b^	<0.001*	46.97±6.680 (46; 32–80)	0.818
	<1 pack a day	132 (15.6)	10.06±4.807 (9; 5–25)^a^		41.70±5.752 (41; 31–60)		47.25±6.936 (47; 28–67)	
	>1 pack a day	59 (7)	9.97±4.382 (9; 5–20)		43.98±6.532 (43; 28–63)^b^		48.12±8.706 (46; 32–80)	
	Smoked and quit	80 (9.4)	10.63±4.193 (10; 5–25)		41.35±6.317 (41; 30–62)		46.85±6.132 (46. 38–68)	
Frequency of tooth- brushing	Once a day	257 (30.5)	10.80±4.422 (10; 5–25)	0.290	40.99±6.539 (40; 28–63)^c^	0.003*	46.41±6.480 (46; 32–68)	0.275
	Twice a day	551 (65.3)	10.87±4.357 (10; 5–25)		40.98±6.376 (40; 23–80)^d^		47.34±6.825 (47; 28–80)	
	1–2 times a week	27 (3.2)	11.52±5.003 (11; 5–21)		41.19±5.711 (43; 28–51)^e^		46.41±6.141 (46; 38–58)	
	Not brushing	60 (1.1)	8.67±4.213 (7; 5–19)		49.33±5.745 (47; 44–62)^c,d,e^		52.56±13.639 (48; 42–80)	
Self–reported oral hygiene	Very good (completely healthy)	52 (6.2)	9.37±3.876 (9; 5–20)	0.022*	44.94±7.795 (44; 32–80)^f,g^	<0.001*	48.73±8.591 (47; 32–80)	0.341
	Good (healthy)	287 (34)	10.55±4.179 (10; 5–25)		40.73±5.989 (40; 28–63)^f^		46.49±6.548 (46; 32–80)	
	Moderate	418 (49.6)	11.07±4.446 (10; 5–25)		40.72±6.606 (40; 23–63)^g^		47.20±6.699 (47; 28–78)	
	Bad (poor)	75 (8.8)	4.446±4.645 (11; 5–25)		41.85±5.654 (41; 28–59)		47.61±7.075 (47; 32–69)	
	Very bad (completely poor)	12 (1.4)	13.50±6.245 (13; 5–21)		40.50±4.275 (41; 35–48)		46.67±7.101 (45.50; 39–59)	
Self–reported oral health	Very good (completely healthy)	45 (5.2)	9.07±3.407 (9; 5–19)^h,i^	0.005*	44.29±8.398 (43; 28–80)^j,k^	0.001*	47.71±9.864 (47; 32–80)	1.000
	Good (healthy)	243 (28.3)	10.39±4.141 (10; 5–25)		41.43±6.371 (41; 28–64)		46.94±6.315 (47; 32–80)	
	Moderate	418 (49.5)	11.07±4.446 (10; 5–25)h		40.56±6.423 (40; 23–63)^j^		47.13±6.955 (46; 28–78)	
	Bad (poor)	116 (13.7)	11.71±4.711 (11; 5–25)		40.58±5.642 (40; 28–62)^k^		46.90±6.012 (47; 36–69)	
	Very bad (completely poor)	24 (2.8)	10.71±5.000 (9.50; 5–21)i		42.88±5.186 (44.00; 28–51)		47.38±6.858 (46; 39–68)	
Frequency of dental visits	Never been	55 (6.5)	11.35±4.54 (12; 5–24)l	<0.001*	42.67±7.93 (43; 28–80)	0.007*	47.13±7.46 (47; 32–80)	0.682
	When a problem occurs	672 (79.6)	11.11±4.40 (10; 5–25)^m^		40.74±6.33 (40; 23–64)^n^		47.07±6.61 (46; 28–78)	
	Regularly	117 (13.9)	9.08±3.86 (8; 5–25)^l,m^		42.31±6.07 (41; 30–62)^n^		47.13±7.73 (46. 32–80)	
Treatments performed in a dental clinic								
Filling	None	377 (44.7)	10.98±4.375 (11; 5–25)	0.273	41.18±6.844 (41; 23–80)	0.779	46.92±7.082 (46;32–80)	0.343
	At least once	467 (55.3)	10.74±4.415 (10; 5–25)		41.00±6.114 (40; 27–63)		47.21±6.616 (47; 28–72)	
Extraction	None	581 (68.8)	10.48±4.219 (10; 5–25)	<0.001*	41.11±6.213 (40; 23–80)	0.560	46.98±6.681 (46; 32–80)	0.500
	At least once	263 (31.2)	11.66±4.671 (11; 5–25)		41.01±6.947 (41; 25–63)		47.32±7.142 (47; 28–80)	
Other	None	536 (63.50)	11.14±4.583 (10. 5–25)	0.029*	40.73±6.528 (40; 25–80)	0.022*	46.95±6.784 (46; 28–80)	0.520
	At least once	308 (36.50)	10.33±4.007 (10; 5–25)		41.69±6.266 (41; 23–64)		47.32±6.902 (47; 32–78)	
*Statistically significant at p<0.05. A statistically significant difference was observed between groups with the same lowercase superscript letters (Dunn’s Test with Bonferroni correction); a: none vs < 1 pack a day, p=0.002; b: none vs > 1 pack a day, p<0.001; c: once a day vs no brushing, p=0.002; d: twice a day vs not brushing, p=0.002; e: 1–2 times a week vs no brushing, p=0.028; f: very good vs good p=0.001, g: very good vs moderate, p<0.001; h: very good vs moderate, p=0.036; i: very good vs very bad, p=0.009; j: very good vs moderate, p=0.006; k: very good vs bad, p=0.039; l: never vs regularly, p=0.001; m: when a problem occurs vs regularly, p<0.001; n: when a problem occurs vs regularly, p=0.020.								

When we compared the MDAS, STAI-1, and STAI-2 anxiety scores, as well as the dental and periodontal status of first- and final-year students, we found statistically significant differences in OHI scores (p<.001) (Table 6).

**Table 6:** Evaluation of MDAS, STAI-1, and STAI-2, DMFT, and OHI status of first and final year students

	First year (n = 255) Mean ± SD (median; min-max)	Final year (n = 333) Mean ± SD (median; min-max)	p
MDAS	11.24±4.392 (11; 5–25)	10.87±4.526 (10; 5–25)	0.194
STAI–1	40.27±6.605 (40; 23–80)	41.68±6.450 (41; 28–64)	0.018
STAI–2	46.48±6.892 (46; 32–80)	47.34±6.922 (46; 28–72)	0.127
D	1.13±1.818 (0; 0–10)	1.05±1.599 (0; 0–10)	0.604
M	0.37±0.891 (0; 0–4)	0.22±0.646 (0; 0–4)	0.063
F	1.61±1.800 (1; 0–9)	1.84±2.22 (1; 0–9)	0.677
DMFT	3.11±2.206 (3; 0–13)	3.10±2.552 (3; 0–13)	0.452
OHİ	0.8787±1.10705 (0.6; 0–6.16)	1.9097±1.83514 (1.1; 0–6)	<0.001*
* Statistically significant at p<0.05, Mann–Whitney U–test.			

A comparison of anxiety levels between first-year and final-year students revealed no statistically significant difference (p=0.324, Pearson’s chi-squared), with the distribution as follows: Respectively, 49.4% and 54.4% of first- and final-year students were considered to have low anxiety (MDAS<10); 43.9% and 37.8% were considered moderately anxious (10-18); and 6.7% and 7.8% of the first- and final-year students were considered to have high or severe anxiety (>18).

When we compared the answers to the MDAS questions between first-year and final-year students, a statistically significant difference was found in MDAS 2 (p=0.013, Pearson’s chi-squared). Regarding “feelings felt while waiting for their turn in the dental office” 75.8 % of the final-year students were found to feel very anxious, whereas 71.4% of first-year students were found to feel extremely anxious (adjusted residual – Iz-skoruI >1.96) (Table 7).

**Table 7:** First and final year students’ answers to MDAS, on a scale of 1 to 5

Questions of MDAS	Response	First year n (%)	Final year n (%)	p
MDAS 1	1	100 (39,2)	155 (46.5)	0.154
	2	111 (43.5)	119 (35,7)	
	3	25 (9.9)	24 (7.2)	
	4	10 (3.9)	18 (5.4)	
	5	9 (3.5)	17 (5.1)	
MDAS 2	1	101 (39.6)	147 (44.1)	0.013*
	2	79 (31)	102 (30.6)	
	3	57 (22.4)	55 (16.5)	
	4	8 (3.1)	25 (7.5)	
	5	10 (3.9)	4 (1.2)	
MDAS 3	1	42 (16.5)	61 (18.3)	0.588
	2	74 (29.0)	111 (33.3)	
	3	81 (31.8)	87 (26.1)	
	4	38 (14.9)	50 (15.0)	
	5	20 (7.8)	24 (7.2)	
MDAS 4	1	91 (35.7)	127 (38.1)	0.233
	2	71 (27.8)	106 (31.8)	
	3	67 (26.3)	62 (18.6)	
	4	20 (7.8)	32 (9.6)	
	5	6 (2.4)	6 (1.8)	
MDAS 5	1	60 (23.5)	82 (24.6)	0.178
	2	73 (28.6)	104 (31.2)	
	3	73 (28.6)	80 (24)	
	4	28 (11.0)	51 (15.2)	
	5	21 (8.2)	16 (4.8)	
* Statistically significant at p<0.05, Mann-Whitney U-test.				

### DISCUSSION

Dental anxiety is a common problem worldwide that often leads individuals to avoid dental treatment. While numerous factors—including psychological and environmental influences—have been identified in the development of dental,^22,32^ previous studies have also suggested that one’s field of education may play a role.^3,21,31^ Based on this opinion, our study aimed to assess the dental anxiety as well as the state and trait anxiety levels of students who are training to become NPH professionals. To our current knowledge, this study is the first to comprehensively evaluate periodontal status, caries prevalence, oral and lifestyle habits, and their association with dental anxiety among non-physician healthcare (NPH) students. The findings may serve as a reference point for understanding oral health-related conditions among healthcare students beyond those enrolled in dentistry and medicine programs.

The prevalence of dental anxiety among university students in various fields varies between 4% and 20%.^27,31^ In our study, the rate of students with excessively high MDAS scores ranged from 6.6% to 7.8% across the academic years, and the overall mean MDAS score was 10.80±4.35. These rates are slightly lower than those reported in previous studies on NPH students.^2,13,21^ Non-dental students typically receive little to no education on oral and dental health in their university program. While a limited number of medical students are exposed to dental medicine and maxillofacial surgery, this remains uncommon. Previous studies comparing anxiety levels among dental, medical, and non-dental students have shown that dental students tend to experience lower levels of anxiety.^3,34^ Although our study focuses on NPH students, our findings reveal anxiety levels comparable to those reported among dental students.

Studies have shown that women exhibit higher scores on the MDAS, STAI-1, and STAI-2 compared to men.^25,27,31^ Parallel to these findings of the aforementioned researchers, our study also revealed that female students demonstrated higher levels of dental anxiety than male students. One possible explanation is that males are generally viewed as more emotionally stable than females.^3,31^ However, other studies have reported no statistically significant difference in dental anxiety between genders.^2,35^ These discrepancies may stem from cultural variations and differences in study populations.

Exposure to proper oral health practices is essential for future healthcare professionals. As students advance through health science programs, their education and clinical experiences can shape their oral health habits. Studying these behaviors before graduation provides valuable insights. Preventive oral health practices include regular tooth brushing and dental visits. In general, it is recommended to brush twice a day.^30^ In our study, 65.3% of the students reported brushing twice a day. This rate is lower than that observed in Croatia (78%),^7^ but higher than in India (48.7%),^19^ and Saudi Arabia (41.9%).^38^ It is also comparable to the findings from Japan (63.2%)^39^ and Turkey (70%)^21^ among non-dental and non-medical educated students. According to our results, the rate of regular dental visits was 13.9%, which is lower than the rates reported in Saudi Arabia (47.7%),^36^ and Japan (28.1%),^39^ yet similar to that observed in Tanzania (14.5%)^29^ among non-dental and non-medical educated students. In the present study, the mean DMFT score was 3.16 (SD ±2.52), which was higher than that reported in India (2.22±2.12),^19^ and Tanzania (1.34±2.44).^29^ The mean OHI score in our study was 1.67 (SD±1.71), which is higher than that examined in Tanzanian nursing students (0.41).^29^

Individuals’ attendance at regular dental check-ups affects their dental anxiety levels.^11,23^ Our study found that MDAS scores were lower in students who attended regular dental check-ups, while state anxiety scores were lower in students who visited the dentist when a problem occurred. These seemingly conflicting results may reflect two different dynamics: regular check-ups may increase awareness and provide them with a greater sense of security, whereas the urgency of pain or discomfort may temporarily overcome dental fear in those seeking immediate relief. Additionally, our study found no statistically significant relationship between anxiety levels and previous dental treatments. In a study investigating the frequency and reasons for visiting the dentist using the Dental Fear Scale, it was found that patients who had never visited a dentist before exhibited higher anxiety.^11^ Similar to this finding, higher dental anxiety was observed in students and adults who visited the dentist irregularly compared to those who visited regularly.^23^

Several studies in the literature have evaluated the dental anxiety levels among dental students throughout their educational journey. These studies commonly report that first-year students tend to experience higher anxiety levels compared to final-year students.^1,27^ In a study, it was reported that preclinical dental hygiene students enrolled in dental hygiene training had higher CDAS scores than their clinical counterparts.^25^ However, in our study, no statistically significant difference was observed in anxiety levels between first- and final-year students, corresponding to the preclinical and clinical years. This inconsistency may be due to differences in educational background. Our participants had not received any oral and dental health education during their undergraduate years. In contrast, the studies mentioned above found that students actively received dental education, which may help to reduce anxiety through increased knowledge, clinical exposure, and hands-on experience as they advance in their training. However, in another study, dental anxiety levels were evaluated using the MDAS among first- and final-year dental students, and no significant difference was found between the groups.^17^ Additionally, in line with previous research, there was no significant difference in terms of DMFT scores between the first and final year;^25^ however, the OHI score increased in the final year of our study.

The findings in our study regarding the relationship between smoking and anxiety (higher MDAS in non-smokers and STAI-1 in smokers) reveal a complex and bidirectional relationship. While there is a general trend in the literature to associate tobacco use with higher dental anxiety, the fact that our findings differ across the MDAS and STAI scales suggests that this relationship is nuanced. The higher MDAS scores in non-smokers can be explained by the automedication hypothesis proposed by some researchers. According to this hypothesis, individuals with high anxiety levels may tend to use cigarettes as a coping mechanism to reduce emotional arousal or distract themselves.^9,20^ Conversely, the higher STAI-1 scores found in smokers may be related to the physiological effects of nicotine and the momentary tension and arousal caused by nicotine withdrawal. The contrast in correlations between these two scales suggests that, despite its suppressive effect on an individual’s trait anxiety level, smoking may negatively impact their momentary physiological state. Therefore, smoking should not be considered a single cause of dental anxiety, but rather an indicator of complex behavioral and physiological interactions.^9,20^

There are limitations to this study. Firstly, this study employed a cross-sectional design, which naturally limits the ability to track changes in dental anxiety over time. As a result, the associations found should not be viewed as cause-and-effect relationships. Secondly, data were gathered through a self-administered questionnaire, which may be subject to response bias. Participants might have hidden their true feelings or underreported their levels of dental fear, anxiety, and discomfort associated with seeking and receiving dental care.

## CONCLUSION

This study concluded that female students, those with low family income, those who describe their oral hygiene as entirely poor, and their oral health as poor have higher dental anxiety. Regular dental visits help reduce anxiety among NPH students. Smoking is also a determining factor in dental anxiety.

Evaluating the dental anxiety of these undergraduates is crucial for identifying educational needs in this field. Also, assessing dental anxiety levels among these students is critical for informing their future professional attitudes toward oral health and for fostering their capacity to motivate patients to pursue dental care. Future studies covering anxiety-reduction programs prompted by workshops and oral-health related class materials, as well as coping strategies are required to reduce dental anxiety among undergraduates.
